# *PD-L1* (*CD274*) and *PD-L2* (*PDCD1LG2*) promoter methylation is associated with HPV infection and transcriptional repression in head and neck squamous cell carcinomas

**DOI:** 10.18632/oncotarget.23080

**Published:** 2017-12-07

**Authors:** Alina Franzen, Timo J. Vogt, Tim Müller, Jörn Dietrich, Andreas Schröck, Carsten Golletz, Peter Brossart, Friedrich Bootz, Jennifer Landsberg, Glen Kristiansen, Dimo Dietrich

**Affiliations:** ^1^ Department of Otolaryngology, Head and Neck Surgery, University Hospital Bonn, Bonn, Germany; ^2^ Institute of Pathology, University Hospital Bonn, Bonn, Germany; ^3^ Department of Oncology, Hematology and Rheumatology, University Hospital Bonn, Bonn, Germany; ^4^ Department of Dermatology, Bonn, University Hospital Bonn, Germany

**Keywords:** PD-L1, PD-1, PD-L2, CD274, PDCD1LG2

## Abstract

**Background:**

DNA methylation of the immune checkpoint gene *PD-L1* has recently been shown to be associated with PD-L1 mRNA expression in various malignancies. This study aimed to investigate the association of *PD-L1* and *PD-L2* methylation with mRNA expression, immune cell infitration, protein expression and human papilloma virus (HPV) infection in head and neck squamous cell carcinoma (HNSCC) patients.

**Results:**

DNA methylation of *PD-L1* and *PD-L2* correlates inversely with mRNA expression (*PD-L1*: *p* ≤ 0.002; *PD-L2*: *p* ≤ 0.014). Methylation of specific CpG-sites of both *PD-L1* and *PD-L2* were further significantly associated with HPV infection in the TCGA cohort. Immune cell infiltrates correlated significantly with *PD-L1* and *PD-L2* methylation. In the validation cohort, PD-L1 protein expression was associated with *PD-L1* hypomethylation (*p* = 0.012).

**Conclusions:**

DNA methylation of *PD-L1* and *PD-L2* is associated with transcriptional silencing and HPV infection in HNSCCs. Additional studies are warranted to test PD-L1 and PD-L2 methylation as predictive biomarkers for response to immunotherapies (e.g. pembrolizumab and nivolumab) that target the PD-L1/PD-L2/PD-1 immune checkpoint axis.

**Materials and Methods:**

*PD-L1* and *PD-L2* promoter methylation and its mRNA expression were analyzed based on Infinium HumanMethylation450 BeadChip and RNA-Seq (both Illumina, Inc.) data in a representative HNSCC patient cohort (*n* = 528) enrolled by The Cancer Genome Atlas (TCGA) Research Network. A validation cohort consisting of 168 HNSCC patients treated at the University Hospital Bonn was analyzed regarding *PD-L1* and *PD-L2* promoter methylation by means of methylation-specific quantitative real-time PCR. PD-L1 protein expression in the validation cohort was quantified via immunohistochemistry (PD-L1 antibody clone 22C3, Dako/Agilent Technologies, Inc.).

## INTRODUCTION

Head and neck squamous cell carcinoma (HNSCC) is a major health burden with over 60,000 newly diagnosed patients in the U.S. every year [1]. The majority of patients presents with either locally advanced or metastatic disease. As a result, the five-year relative-survival rate is only around 64% [1]. HNSCCs represent a heterogeneous group of tumors with distinct etiology, clinical behaviour and treatment responses [2]. Tobacco and alcohol abuse and high-risk types of the human papilloma virus (HPV) are major risk factors for HNSCC resulting in different genetic and epigenetic subtypes [3, 4]. This complexity necessitates predictive biomarkers allowing for the optimization of individualized treatment protocols. Therapeutic strategies include radical surgery as well as radiation, targeted therapies with tyrosine kinase inhibitors (cetuximab) and concomitant chemotherapy, the latter agents producing significant toxicity at therapeutic doses. Recently, immunotherapy has increasingly gained attention as a novel therapeutic option for HNSCC. Studies on the interaction between tumor and host immune response have been focusing particularly on the programmed death-1 receptor (PD-1) and its ligand PD-L1 (programmed death-1 ligand) pathway as potential immunotherapeutic target [5–9].

Treatment with the PD-1 targeting antibodies pembrolizumab (Keytruda^®^, Merck & Co., Inc., US) or nivolumab (Opdivo^®^, Bristol-Myer Squibb, US) achieved durable antitumor activity in recurrent and/or metastatic HNSCC. The KEYNOTE-012 and Checkmate 141 trials have revealed overall response rates of 18% and 13.3%, respectively [6, 7]. Of note, an exploratory analysis of the Checkmate 141 trial published this year revealed nivolumab delaying the time to decimation of patient’s quality-of-life compared to monotherapy of investigator’s choice in patients with platinum-refractory recurrent or metastatic HNSCC [8]. Furthermore, also pembrolizumab exhibits an acceptable toxicity profile in recurrent/metastatic HNSCC previously treated with platinum and cetuximab [10]. However, the phase III KEYNOTE-040 trial recently failed to meet the primary endpoint of overall survival in patients with previously treated recurrent or metastatic HNSCC [11]. Thus, predictive biomarkers that allow for the identification of patients that are likely to respond represent an urgent medical need.

Transcription of the gene *CD274* coding for PD-L1 is constitutively upregulated in solid tumors, including HNSCC [12]. PD-L2 encoded by the gene *PDCD1LG2* is a second ligand for PD-1 that inhibits T cell activation [13]. PD-L1 and PD-L2 bind PD-1 with similar affinities, but with significantly different association and dissociation characteristics [14]. PD-L2 has not received as much attention compared to PD-L1 and its specific role in modulating tumor immunity is less clear. Via binding its ligands PD-L1 and PD-L2, the PD-1 receptor initiates a reduction of T cell receptor activity and elicits immunoevasion [14]. Tumor biology implies that only PD-L1-positive tumors are likely to respond to therapy with PD-1 antagonists. Indeed, expanded investigations in multiple solid tumor entities including melanoma, non-small cell pulmonary carcinoma, bladder cancer, and renal cell carcinoma have validated this general concept [for review: 5, 9]. Of note, however, subsequent studies have also revealed a lower but finite response rate in patients with tumors devoid of PD-L1 expression, calling into question the use of PD-L1 protein expression as an absolute selection criterion for therapy [6, 7, for review: 5, 9]. The more and more widespread use of immune targeted therapies brings about that novel biomarkers are urgently needed to assist guiding patient selection and providing early on-treatment indicators of response. Recent studies suggest epigenetic control via DNA methylation likely to play a fundamental role within the dynamic expression of the PD-1/PD-L1 checkpoint axis [15–21]. In HNSCC, we recently showed that promoter methylation of the PD-1 encoding gene *PDCD1* is associated with HPV infection and poor prognosis [20]. In the present study, we aim at elucidating the impact of DNA methylation within the *CD274*/*PD-L1* and *PDCD1LG2*/*PD-L2* genes on the respective gene expression and the association with HPV infectionin HNSCC specimens from a large multicentre cohort (provided by The Cancer Genome Atlas Research Network) and a small validation cohort from the University Hospital Bonn.

## RESULTS

### *PD-L1* and *PD-L2* is hypomethylated in tumor compared to normal adjacent tissue

**Figure 1:**
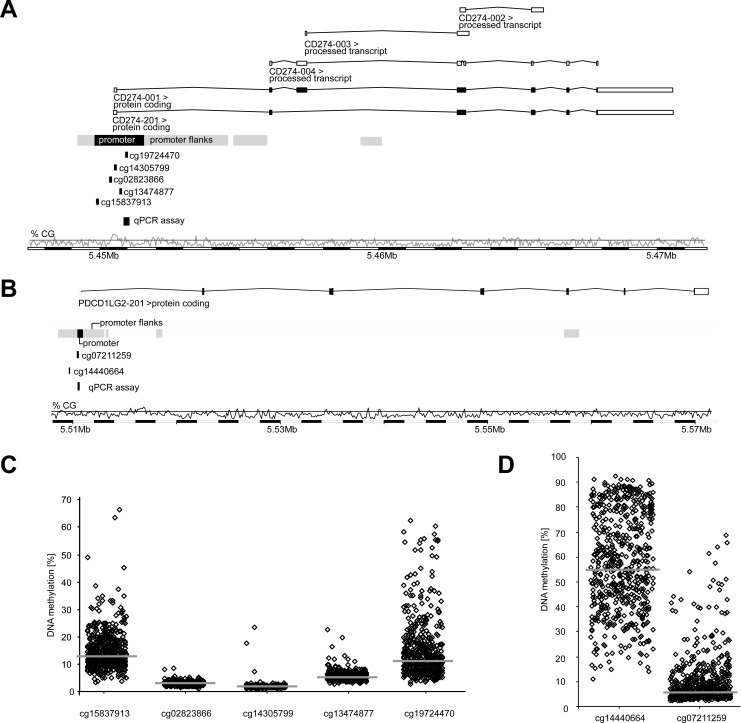
Organization and promoter methylation of the *PD-L1* (*CD274*) and *PD-L2* (*PDCD1LG2*) genes Organization of *PD-L1* (**A**) and *PD-L2* (**B**) genes, location of Illumina Infinium HumanMethylation450 BeadChip beads, qPCR assays, and CG-density of the gene region. Shown are relative and median (indicated in bars) m*PD-L1* (**C**) and m*PD-L2* (**D**) levels obtained for each single bead in the HNSCC TCGA cohort (*n =* 528). Beads targeting peripheral CpG-sites of the respective promoter regions reveal higher methylation levels (14.7 ± 7.10% for cg1537913 and 14.9 ± 10.7% for cg19724470 (all C); 56.3 ± 19.1% for cg14440664 (D)) than those targeting central CpG-dense areas (3.15 ± 7.35% for cg02823866; 2.08 ± 1.22% for cg14305799; 5.84 ± 1.71% for cg13474877 (all C); 8.98 ± 9.11% for cg07211259 (D)).

For the analysis of *PD-L1* promoter methylation within the TCGA cohort, Illumina Infinium HumanMethylation450 BeadChip beads (for *PD-L1*: cg15837913, cg02823866, cg14305799, cg13474877, cg19724470 and for *PD-L2*: cg14440664 and cg07211259) targeting loci within the promoter regions of the *PD-L1* or *PD-L2* genes were used (Figure 1A and 1B). CpG-sites targeted by beads cg15837913 (median methylation: 13.0%) and cg19724470 (median methylation: 11.4%), both sites located peripheral in the CpG-dense area of *CD274*, showed higher methylation levels than those beads in central position of the CpG-dense area (median methylation cg02823866: 3.10%, cg14305799: 1.96%, cg13474877: 5.57%; Figure 1C). This was similar for *PD-L2* which showed higher methylation levels at the more peripherally located target region of bead cg14440664 (median methylation: 55.1%) compared to cg07211259 (median methylation: 6.0%, Figure 1D). Interestingly, hypomethylation was found in tumors compared to normal adjacent tissues (NATs) at both *PD-L2* (*p <* 0.001) and four out of five *PD-L1* (*p ≤* 0.047, Table 1) gene loci analyzed. In contrast, the most centrally located bead cg14305799 revealed higher methylation in tumors as compared to NAT (*p =* 0.001). While PD-L2 mRNA expression was significantly higher (*p <* 0.001) in tumors, PD-L1 mRNA expression showed no difference.

**Table 1:** Association of *PD-L1* and *PD-L2* methylation with mRNA expression and HPV-status

Analyte	Median methylation [%] and mRNA expression [normalized counts]			Correlation with *PD-1* methylation^†^		Correlation with PD-1 mRNA expression^†^		Correlation with PD-L1 mRNA expression^†^		Correlation with PD-L2 mRNA expression^†^		Median methylation [%] and mRNA expression [normalized counts]		
	Tumor	NAT	*p*-value^‡^	Spearman’s ρ	*p*-value	Spearman’s ρ	*p*-value	Spearman’s ρ	*p*-value	Spearman’s ρ	*p*-value	HPV-neg.	HPV-pos.	*p*-value^‡^
PD-L1 mRNA	80.6	54.0	0.31	0.063	0.15	0.520	<0.001	NA	NA	0.761	<0.001	82.3	88.0	0.42
PD-L2 mRNA	98.0	47.6	<0.001	0.104	0.018	0.491	<0.001	0.761	<0.001	NA	NA	115.6	67.1	0.023
m*PD-L1* (cg15837913)	13.0	17.3	<0.001	0.106	0.015	–0.314	<0.001	–0.320	<0.001	–0.197	<0.001	13.9	11.1	0.051
m*PD-L1* (cg02823866)	3.10	3.31	0.026	–0.053	0.22	–0.077	0.078	–0.133	0.002	–0.107	0.014	3.20	3.35	0.23
m*PD-L1* (cg14305799)	1.96	1.85	0.001	–0.026	0.55	–0.150	0.001	–0.183	<0.001	–0.199	<0.001	1.93	2.05	0.28
m*PD-L1* (cg13474877)	5.57	5.94	0.047	–0.017	0.70	–0.250	<0.001	–0.327	<0.001	–0.241	<0.001	5.84	5.70	0.82
m*PD-L1* (cg19724470)	11.4	17.0	<0.001	0.120	0.006	–0.240	<0.001	–0.444	<0.001	–0.322	<0.001	12.6	18.0	0.011
m*PD-L2* (cg14440664)	55.1	80.1	<0.001	–0.053	0.22	0.283	<0.001	0.007	0.87	–0.176	<0.001	53.8	82.1	<0.001
m*PD-L2* (cg07211259)	6.0	13.0	<0.001	0.240	<0.001	–0.174	<0.001	–0.160	<0.001	–0.153	<0.001	6.42	6.45	0.61

DNA methylation of the *PD-L1* and *PD-L2* gene loci and correlation/association with PD-L1 and PD-L2 mRNA expression, *PD-1* methylation and PD-1 mRNA expression, and HPV-status. DNA methylation of the *PD-L1* and *PD-L2* gene loci were determined at five and two positions (Figure 1), respectively, within the promoter regions. Methylation data, mRNA expression data, and HPV status were available for *n* = 528 tumors and *n* = 50 NATs (methylation), *n* = 520 tumors and *n* = 50 NATs (mRNA), and *n* = 279 tumors (HPV), respectively. *PD-1* methylation data were adopted from Goltz *et al.* [19] and HPV-status as determined via RNAseq (*n* = 36 HPV-positive, *n* = 243 HPV-negative tumors) was extracted from The Cancer Genome Atlas Research Network [3]. ^‡^Mann-Whitney *U* test, ^†^Spearman’s correlation; NA: Not Applicable.

**Figure 2:**
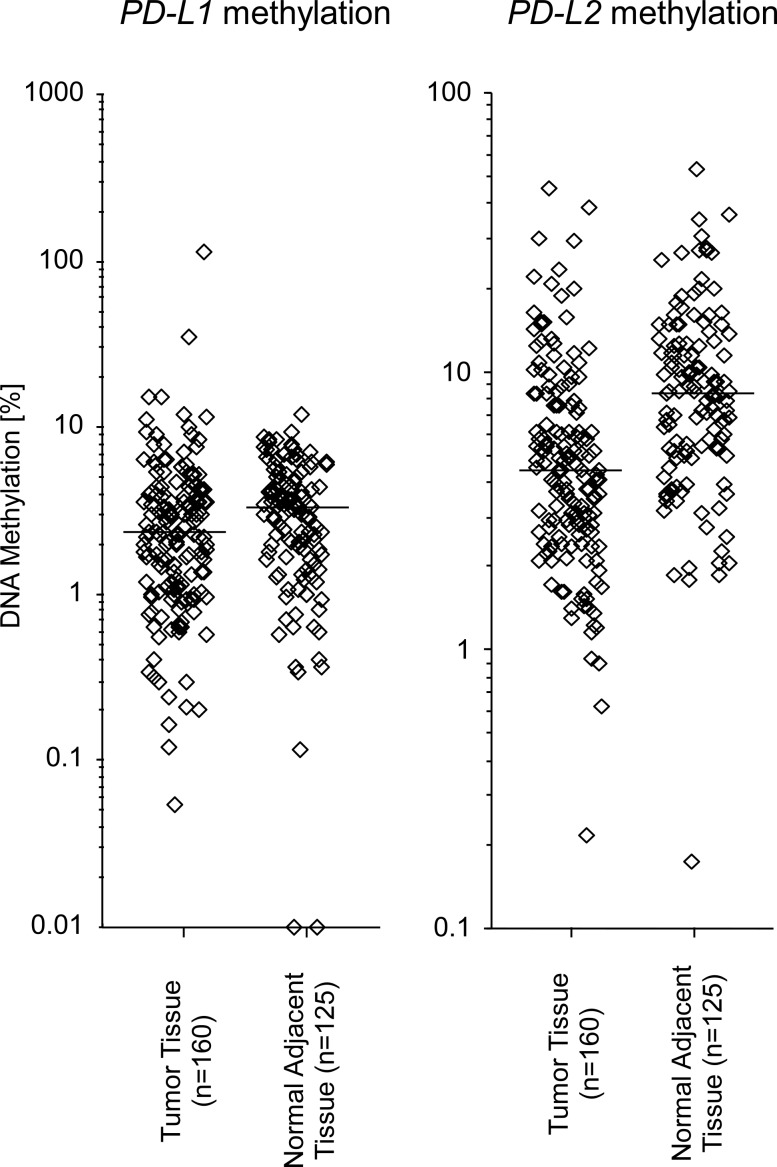
*PD-L1* and *PD-L2* methylation in HNSCC and normal adjacent tissue in the UKB cohort *PD-L1* and *PD-L2* methylation in 160 HNSCC and 125 NAT. Mean *PD-L1* methylation levels were not significantly different in tumors versus NATs (*PD-L1* median methylation in tumors: 2.34%, NATs: 3.28%, *p =* 0.077, Mann–Whitney *U* test; *PD-L2*, median methylation in tumors: 4.42%, NATs: 8.32%, *p <* 0.001, Mann–Whitney *U* test). Bars indicate median methylation levels.

The analysis of 161 tumor tissues and 126 NATs from the UKB cohort confirmed *PD-L2* hypomethylation in tumors compared to NATs (median methylation in tumors: 4.42%, NATs: 8.32%, *p <* 0.001, Mann–Whitney *U* test, Figure 2). *PD-L1* methylation levels only showed a trend towards lower methylation in tumors versus NATs (median methylation in tumors: 2.34%, NATs: 3.28%, *p =* 0.077, Mann-Whitney *U* test).

The correlations and associations of *PD-L1* and *PD-L2* promoter methylation with clinicopathologic variables in both cohorts can be found in Supplementary Table 1 and Supplementary Table 2.

### *PD-L1* and *PD-L2* promoter methylation correlates inversely with mRNA expression

All seven Illumina Infinium beads targeting the promoters of *PD-L1* and *PD-L2* showed a significant inverse correlation with the respective PD-L1 and PD-L2 mRNA expression (Table 1). This finding indicates an epigenetic regulation mechanism of the *PD-L1* and *PD-L2* genes.

The correlations and associations of PD-L1 and PD-L2 mRNA expression with clinicopathologic variables in the TCGA cohort can be found in Supplementary Table 1.

### *PD-L1* and *PD-L2* promoter methylation correlates with *PD-1* methylation and mRNA expression

We have earlier reported *PD-1* methylation as strong prognostic parameter that is associated with HPV infection in HNSCC [20]. This prompted us to investigate the correlation between *PD-1* methylation and expression with *PD-L1* and *PD-L2* methylation and mRNA expression. *PD-1* methylation correlated positively with PD-L2 mRNA expression and methylation of *PD-L1* and *PD-L2* loci targeted with beads cg15837913, cg19704470 and cg07211259 (Table 1). Correspondingly, we found a negative correlation of PD-1 mRNA expression with PD-L1 and PD-L2 methylation at five loci, however, a significant positive correlation is present at *PD-L2* locus targeted via bead cg14440664.

### *PD-L1* promoter methylation correlates inversely with protein expression

To date, no research exists that firmly establishes a connection between *PD-L1* methylation and PD-L1 protein expression. The latter correlation was investigated in the UKB cohort only. Matched methylation levels and PD-L1 expression data were obtained from 146 tumors. *PD-L1* methylation was dichotomized according to its median and groups referred to as m*PD-L1*_low_ and m*PD-L1*_high._ PD-L1-positive tumors were significantly more often assigned to the m*PD-L1*_low_ subgroup than to the than m*PD-L1*_high_ subgroup (*Χ*^2^ = 6.25, *p =* 0.012). Clinicopathological correlations and associations of PD-L1 protein expression in the UKB cohort are found in Supplementary Table 2.

### *PD-L1* and *PD-L2* promoter methylation is associated with HPV infection

**Figure 3:**
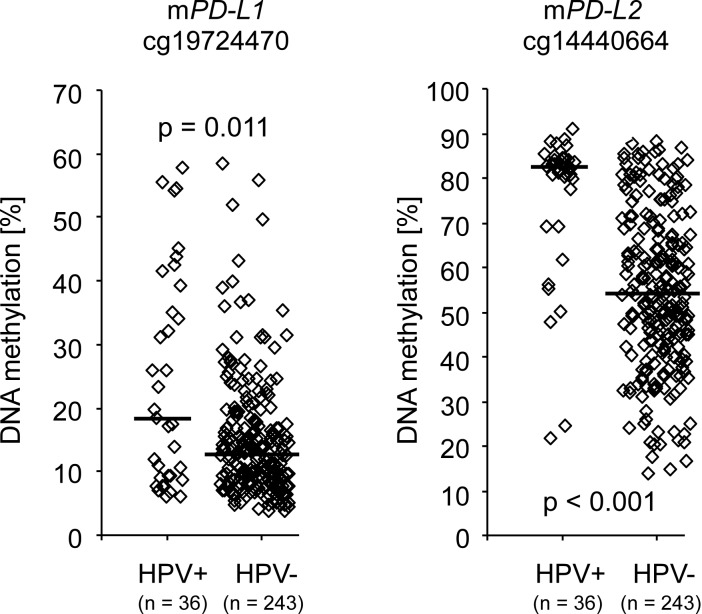
*PD-L1/PD-L2* methylation and HPV-status Differential *PD-L1/PD-L2* DNA methylation (cg19724470 and cg14440664) stratified according to HPV-status analyzed in 279 individuals with procurable HPV-status of the TCGA cohort. Bars indicate median methylation levels. *P*-values refer to Mann–Whitney *U* test.

The HPV status of a subgroup of 279 tumors from the TCGA cohort was reliably determined via RNASeq and 243 tumors were identified as HPV-negative and 36 as HPV-positive [3]. Significantly higher *PD-L1* and *PD-L2* methylation levels in HPV-positive compared to HPV–negative tumors were found at loci targeted by beads cg19724470 (*PD-L1*) and cg14440664 (*PD-L2*) (*p <* 0.011, Table 1, Figure 3). In contrast, a trend (*p =* 0.051) towards lower methylation in HPV-positive compared to –negative tumors was found for *PD-L1* bead cg15837913. PD-L2 mRNA expression was lower in HPV-positive compared to –negative tumors (*p =* 0.023, Table 1) while no significant difference was found with regard to PD-L1 mRNA expression.

p16 status as a surrogate for HPV infection was available for 133 patients of the UKB cohort (100 p16-positive, 33 p16-negative). However, an association of the *PD-L1* and *PD-L2* methylation with p16-status in the UKB cohort could not be found (*PD-L1*: *p =* 1.0, *PD-L2*: *p =* 0.24, Mann–Whitney *U* test). The *PD-L2* qPCR assay targets the binding region of bead cg07211259 which already did not reveal methylation differences between HPV-negative and HPV-positive tumors in the TCGA cohort (Table 1). The *PD-L1* qPCR hydrolysis probe, however, assays the target region of bead cg19724470 and therefore could not confirm the finding from the TCGA cohort.

### *PD-L1* and *PD-L2* promoter methylation is associated with immune cell infiltrates

Additionally, immune cell infiltrates (B, T, and dendritic cells) were correlated with *PD-L1*/*PD-L2* methylation and PD-L1/PD-L2 mRNA expression in the TCGA cohort. PD-L2 and PD-L1 mRNA expression was significantly positively correlated with all analyzed immune cell infiltrates (Table 2). The strongest correlations were found between PD-L1/PD-L2 mRNA expression and T cell (CD4^+^ and CD8^+^) and dendritic cells while the correlation with B cell infiltrates was only weak. Significant inverse correlation between *PD-L1* methylation and infiltrates of dendritic and CD8^+^ T cells (cg15837913, cg14305799, cg13474877, cg19724470) and CD4^+^ T cells (cg14305799 and cg13474877) were observed. No correlation was found between *PD-L1* methylation and infiltration of B cells. In contrast, a strong positive correlation of *PD-L2* methylation (bead cg14440664) with B cell infiltrates was observed. While *PD-L1* methylation was inversely correlated with T and dendritic cell infiltrates, *PD-L2* methylation as determined with the HPV-associated locus targeted by bead cg14440664 showed a strong positive correlation with the infiltration of all immune cells under investigation (Table 2).

**Table 2:** Correlation of *PD-L1* and *PD-L2* methylation and mRNA expression with immune cell infiltrates

Analyte	B cells		T cells (CD4^+^)		T cells (CD8^+^)		Dendritic cells	
	Spearman’s ρ	*p*-value	Spearman’s ρ	*p*-value	Spearman’s ρ	*p*-value	Spearman’s ρ	*p*-value
PD-L1 mRNA	0.086	0.051	0.359	<0.001	0.420	<0.001	0.545	<0.001
PD-L2 mRNA	0.093	0.036	0.398	<0.001	0.399	<0.001	0.624	<0.001
cg15837913 (*PD-L1*)	0.024	0.59	0.007	0.88	**–**0.197	<0.001	**–**0.086	0.052
cg02823866 (*PD-L1*)	0.093	0.036	0.010	0.82	**–**0.020	0.64	**–**0.020	0.65
cg14305799 (*PD-L1*)	-0.071	0.11	**–**0.111	0.012	**–**0.122	0.006	**–**0.112	0.011
cg13474877 (*PD-L1*)	-0.034	0.44	**–**0.079	0.073	**–**0.166	<0.001	**–**0.129	0.003
cg19724470 (*PD-L1*)	0.099	0.025	**–**0.010	0.83	**–**0.136	0.002	**–**0.079	0.073
cg14440664 (*PD-L2*)	0.394	<0.001	0.340	<0.001	0.326	<0.001	0.275	<0.001
cg07211259 (*PD-L2*)	0.091	0.040	0.053	0.23	**–**0.065	0.14	**–**0.008	0.86

Spearman’s correlation (ρ, *p*-value) between *PD-L1* and *PD-L2* promoter methylation together with PD-L1 and PD-L2 mRNA expression and immune cell infiltrates. Immune cell infiltrates were determined using RNA-Seq based quantification as shown by Li *et al.* [22].

## DISCUSSION

Recently, the PD-1 checkpoint inhibitors nivolumab and pembrolizumab that block the binding of the PD-1 ligands PD-L1 and PD-L2 have gained regulatory approval for the treatment of recurrent and metastasized HNSCC. There is an evolving body of evidence suggesting that PD-L1 expression on the surface of tumor cells themselves and on tumor infiltrating immune cells may contribute to outcome in HNSCC and other solid tumors under immune checkpoint inhibition. Immunohistochemical assessment of intratumoral PD-L1 protein expression has been used to predict the response to PD-1/PD-L1 immune checkpoint blockage in several solid tumor entitiesfrom early therapeutic attempts on [5]. Subsequently, its utility has been stressed by the accreditation of PD-L1 diagnostic biomarker tests targeting different PD-L1 epitopes by immunohistochemical staining [for review: 5, 9]. However, PD-L1 positivity has been inconsistently defined in clinical studies ranging from 1% up to 50% of cells expressing PD-L1 [reviewed in: 9]. Huge clinical ‘intent-to-treat’ studies with immune checkpoint inhibitors, however, reported on clinical benefit in biomarker-unselected patients with HNSCC [6, for review: 9]. Notably, the number of clinical trials has been steadily increasing over the last years observing finite response rates in patients with PD-L1 negative tumors [for review: 9], constituting key observations that potentially point to gaps in the utility of PD-L1 protein expression as a predictive biomarker that will necessarily be addressed in the future.

A potential pitfall of PD-L1 immunohistochemical tests is based on the fact that expression of PD-L1 may vary greatly in a single pre-treatment tumor specimen, which may indeed be due to a high turnover of membranous PD-L1 protein. It seems crucial to recall that adaptive expression in response to pro-inflammatory factors on the tumor surface may add to heterogeneous PD-L1 protein expression in tumors that lack constitutive activation by innately dysregulated signaling pathways. This may especially hold true for HNSCC, since PD-L1 expression has been shown to involve both constitutive and adaptive mechanisms in HNSCC [23]. While PD-L1 protein expression is heterogeneous in terms of spatiotemporal distribution, epigenetic modification of DNA may be more stable and therefore more robustly detectable in routine diagnostics. An inverse correlation between promoter methylation and mRNA expression levels as well as protein expression implies a technically and biologically robust measure and suggested that the level of PD-L1 expression might in part be inflicted by epigenetic modification in epithelial derived tumors.

We have shown recently, that *PD-1* methylation in HNSCC is associated with survival and HPV infection in HNSCC, indicating an epigenetic regulation of the PD-1/PD-L1/PD-L2 immune checkpoint axis in HNSCC [20]. The present study clearly showed an inverse correlation between *PD-L1* and *PD-L2* promoter methylation with the respective mRNA expression, supporting the hypothesis of DNA methylation as an epigenetic silencing mechanism of these immune checkpoint genes in HNSCC. From our point of view, the present study will be of considerable value demonstrating that membranous PD-L1 protein expression may be traced back to differential *PD-L1* methylation in HNSCC. In this respect, quantitative methylation analysis seems to be a groundbreaking technical extension potentially offering automated processes to determine immunoresponsiveness in HNSCC. From the oncologist’s point of view, what is needed are biomarkers which can be quickly implemented and robustly determine the individual chance of response.

Our study suffers from two major limitations. While we were able to show a general inverse correlation between methylation and mRNA expression for all analyzed CpG-sites within the respective gene in the TCGA cohort, the association with HPV infection seems to be much more nuanced and restricted to certain single CpGs. Hence, a more detailed analysis of each single CpG-site is required. This finding also might be an explanation for the discrepant results obtained with the Illumina bead chip and quantitative real-time PCR technologies, which both target different CpG-sites. Bead cg19724470 for example targets two CpG-sites, while the respective *PD-L1* qPCR probed 5 CpG sites of which the two CpG-sites included into the bead cg19724470 are only targeted by the qPCR hydrolysis probe and not by the primers. The same limitation applies to the *PD-L2* qPCR assay which targets three CpG-sites including the single CpG-sites probed by the respective bead cg07211259. Accordingly, a quantitative approach that allows for methylation analysis at single CpG-site resolution, e.g. quantitative bisulfite sequencing [24], is required to identify those CpG-sites that are most significantly associated with HPV infection and transcriptional repression. A second limitation is the lack of a cell type-specific methylation and expression analysis. Regarding *PD-L2* methylation, a strong positive correlation with immune cell infiltrates, PD-1 expression and HPV-related hypermethylation is found at locus targeted by bead cg14440664. Kadel *et al.* [25] identified different methylation in peripheral blood leukocytes compared to tumor cells which is most profound at the border region of the CpG-dense area within the promoter. Consequently, methylation at *PD-L2* locus cg14440664 might originate from immune cells which specifically infiltrate HPV-positive tumors. This would be in line with distinct patterns of HPV-related immune cell infiltrates [26, 27]. However, median methylation at this specific gene locus is 80.1% in normal adjacent tissues and consequently is unlikely to originate from immune cell nucleic DNA only. On the other hand, HPV has shown to be associated with a distinct methylation phenotype [4] which might be accompanied with the infiltration of specific immune cells. Thus, a detailed analysis of the methylation at single CpG-site resolution in distinct cell types present in the tumor is required to understand the interaction between methylation, HPV infection and immune response.

Based on our evolving knowledge of the underlying pathways, PD-L1 immunohistochemical testing in the context of therapies with PD-1/PD-L1 antagonists only in part meets the demands on a predictive biomarker. Gene methylation, on the other hand, seems to be a favored candidate for a predictive biomarker in that it can accurately and robustly be determined in various sample types, including minute amounts of formalin-fixed and paraffin-embedded tissues [28–30]. Recently, we were able to show that paired analysis of small biopsy specimens and gross surgical resections results in concordant methylation values [31]. We believe that *PD-L1* and *PD-L2* methylation warrants further evaluation in the context of prospective studies as a biomarker for response prediction to treatments with anti-PD-1/PD-L1 antibodies, i.e. pembrolizumab and nivolumab.

## MATERIALS AND METHODS

The results shown here are partly based upon data generated by The Cancer Genome Atlas (TCGA) Research Network: http://cancergenome.nih.gov/.

### Patient cohorts

#### TCGA cohort

The TCGA patient cohort is comprised of 528 retrospectively enrolled HNSCC patients. Informed consent was acquired from all patients in accordance with the Helsinki Declaration of 1975 by the TCGA Research Network. In addition to surgery, patients received neoadjuvant therapy (TCGA variable „history_of_neoadjuvant_treatment“, yes: *n =* 10, no: *n =* 518), adjuvant postoperative radiation therapy (TCGA variable „radiation_therapy“, yes: *n =* 126, no: *n =* 64, unknown/not available: *n =* 338), and adjuvant postoperative pharmacotherapy (TCGA variable „postoperative_rx_tx“, yes: *n =* 66, no: *n =* 121, unknown/not available/discrepant: *n =* 341). The TCGA data set provides data on 36 HPV-driven HNSCC, 243 HNSCC that occurred without HPV infection as well as 50 samples of normal adjacent tissue (NAT). Tumor samples where classified as HPV-positive or -negative by mapping of RNASeq reads [3]. *PD-L1* and *PD-L2* promoter methylation was assessable for 528 specimens. *PD-L1* and *PD-L2* mRNA expression data were available from 520 patients.

#### University hospital bonn (UKB) cohort

168 HNSCC patients who underwent surgical resection at the University Hospital Bonn between 2010 and 2014 were retrospectively enrolled. The study protocol was approved by the ethics committee of the University Hospital Bonn. *PD-L1* and *PD-L2* promoter methylation was assessable for 160 tumor and 125 NAT samples. PD-L1 protein expression determined via immunohistochemistry (IHC) was available from 157 samples.

### DNA methylation analysis

Infinium HumanMethylation450 BeadChip (Illumina, Inc., San Diego, CA, USA) data of level 2 were downloaded from the TCGA webpage. Background-corrected unmethylated (intensity_U) and methylated (intensity_M) summary intensities as extracted by means of the R package ‘methylumi’ were applied. Methylation of the *PD-L1* (m *PD-L1*) and *PD-L2* (m *PD-L2*) promoter regions HNSCC patient samples was analyzed using seven Illumina Infinium HumanMethylation450 BeadChip beads (*PD-L1*: cg15837913, cg02823866, cg14305799, cg13474877, and cg19724470; *PD-L2*: cg14440664 and cg07211259), which are located in the *PD-L1* and *PD-L2* promoter region (Figure 1). Methylation levels for each of the seven beads were calculated by the formula: 100% × intensity_M/(intensity_M + intensity_U).

Bisulfite-converted DNA from the UKB cohort was prepared using the innuCONVERT Bisulfite All-In-One Kit (Analytik Jena AG, Jena, Germany) [32] following the manufacturer’s instructions. Quantitative methylation-specific real-time PCR to quantify m*PD-L1* was performed as described previously [15]. In brief, an assay comprised of methylation-specific primers and a methylation-specific hydrolysis probe (probe: 6-FAM-cacgaatccaaatccaccgccaac-BHQ-1; reverse primer: cgtttagggattttggatttgtttagc; forward primer: atataaaataaataatcattcttatacg) targeting the region Chr9:5450860-5451050 (GRCh38.p10) within the *PD-L1* promoter was duplexed with an assay specifically amplifying a CpG-free region within the *ACTB* gene locus. The target region of this *PD-L1* assay overlaps with the target region of the peripheral bead cg19724470 [15]. m*PD-L2* was assessed using a quantitative methylation-specific real-time PCR assay targeting the locus Chr9:5510477-5510616 (GRCh38.p10) that overlaps with the target region of the central bead cg07211259. PCR composition and cycling program was identical as for *PD-L1* assay but with *PD-L2*-specific oligonucleotides (forward primer: ttttaaataagttaggttttcgtt; reverse primer: aaaaaacactcaaaatttaacgt; hydrolysis probe: 6-FAM-ttatttttatgttacggtaaattttaa-BHQ-1). 20 ng bisulfite-converted template DNA quantified via UV-spectrophotometry was measured in triplicate. Quantitative DNA methylation levels were calculated using the ΔΔCT method adapted for methylation analyses [28].

### mRNA expression analysis

RNA-Seq Version 2 data (normalized counts) were generated by means of the Illumina HiSeq 2000 RNA Sequencing Version 2 analysis (Illumina, Inc., San Diego, CA, USA) and obtained from the TCGA Research Network.

### Protein expression analysis

IHC quantification of PD-L1 protein expression was performed using the PD-L1 antibody clone 22C3 (Dako/Agilent Technologies, Inc., Santa Clara, CA, USA) according to the manufacturer’s instructions. PD-L1 expression was regarded as positive in cases with ≥1% positive membranous staining.

### Examination of tumor infiltrating immune cells

Quantitative data on immune cell infiltrates (B cells, CD4^+^ and CD8^+^ cells, macrophages, and dendritic cells) were obtained from Li *et al.* [22] and were available from 514 patients’ samples.

### Statistical analysis

The statistical analyses were performed using SPSS, version 23 (SPSS Inc., Chicago, IL). Correlations between m*PD-L1*/2 and PD-L1/2 mRNA expression were analyzed using the Spearman’s rank correlation (Spearman’s ρ). Differences between groups were tested using Mann-Whitney *U* test, Kruskal-Wallis test, *Χ*^2^ test, *t*-test and ANOVA with Bonferroni post hoc analysis. *P*-values < 0.05 were considered to be statistically significant.

## SUPPLEMENTARY MATERIALS FIGURES AND TABLES






