# Supplementary material for: PD-L1 (CD274) and PD-L2 (PDCD1LG2) promoter methylation is associated with HPV infection and transcriptional repression in head and neck squamous cell carcinomas
Source: Oncotarget. 2017 Dec 7;9(1):641–50. doi: 10.18632/oncotarget.23080 (PMC5787495; doi:10.18632/oncotarget.23080)
Supplement: Supplementary file 1 [file oncotarget-09-641-s001.pdf]

PD-L1 (*CD274*) and *PD-L2* (*PDCD1LG2*) promoter methylation is associated with HPV infection and transcriptional repression in head and neck squamous cell carcinomas

SUPPLEMENTARY MATERIALS

Supplementary Table 1: TCGA Cohort. See Supplemntary_Table_1

Supplementary Table 2: UKB Cohort. See Supplemntary_Table_2
